# Crops under stress: can we mitigate the impacts of climate change on agriculture and launch the ‘Resilience Revolution’?

**DOI:** 10.1098/rstb.2024.0228

**Published:** 2025-05-29

**Authors:** Ron Mittler, Rumyana Karlova, Diane C. Bassham, Tracy Lawson

**Affiliations:** ^1^ University of Missouri, Columbia, MO 65201, USA; ^2^ Laboratory of Plant Physiology, Plant Science Group, Wageningen University & Research, Wageningen 6708, The Netherlands; ^3^ Iowa State University, Ames, IA 50011, USA; ^4^ School of Lif Sciences, University of Essex Faculty of Science and Engineering, Colchester CO4 3SQ, UK

**Keywords:** climate change, agriculture, stress, crop, yield, breeding

## Abstract

Climate change is altering our environment, subjecting multiple agroecosystems worldwide to an increased frequency and intensity of abiotic stress conditions such as heat, drought, flooding, salinity, cold and/or their potential combinations. These stresses impact plant growth, yield and survival, causing losses of billions of dollars to agricultural productivity, and in extreme cases they lead to famine, migration and even wars. As the rate of change in our environment has dramatically accelerated in recent years, more research is urgently needed to discover and develop new ways and tools to increase the resilience of crops to different stress conditions. In this theme issue, new studies addressing the molecular, metabolic, and physiological responses of crops and other plants to abiotic stress challenges are discussed, as well as the potential to exploit these mechanisms in biotechnological applications aimed at preserving and/or increasing crop yield under our changing climate conditions.

This article is part of the theme issue ‘Crops under stress: can we mitigate the impacts of climate change on agriculture and launch the ‘Resilience Revolution’?’

## 1. Introduction

Global warming and climate change, along with the extreme weather patterns they impose on different areas of our planet, are among the most severe existential threats currently faced by humanity [1–4]. When compared with pre-industrial revolution conditions, the severity and frequency of droughts, heat waves, storms, floods, cold snaps and freezing episodes have dramatically increased in recent years [1,5]. These occur in the background of deteriorating soil conditions that include enhanced salinity, microplastics content and pH extremes, as well as reduced microbiome diversity, which are also considered an outcome of modern industrial and agricultural practices [6–9]. In some instances, for example when a heat wave occurs during a drought or following a flood, plants and crops are further subjected to a combination of two or more different abiotic stress factors, simultaneously or sequentially [10–12]. The combined effects of altered weather patterns and weakening of plants are also thought to result in substantial outbreaks of diseases and/or insect attacks [5]. Taken together, these adverse abiotic and biotic conditions impact crops and other plants, causing a decrease in growth, reproduction, yield and even plant survival, inflicting massive losses to agricultural productivity, and potentially leading, under extreme circumstances, to famine, migration, wars, and the overall destabilization of different societies worldwide [1,4,13,14].

**Figure 1.**
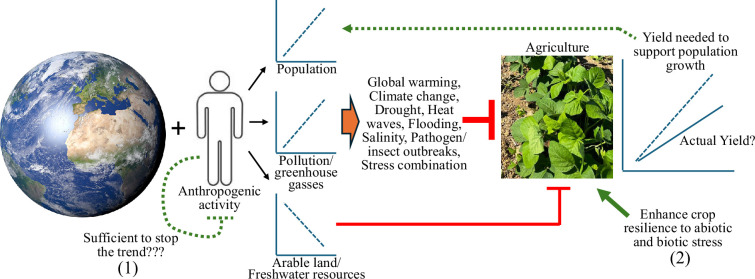
The impacts of anthropogenic activities on agriculture and their negative effects on yield and food supply. The increase in human population coupled with the increased levels of pollution and greenhouse gasses that humans produce (a major driver of global warming and climate change that cause an increase in droughts, heat waves, flooding, etc.) and the decrease in prime agricultural land and freshwater resources are negatively impacting agricultural production to the point that it may not be able to support the existing and/or projected human population in the coming years. If not stopped, this process will destabilize our society and cause hunger, poverty, migration and even wars. Two main processes need to be altered if we want to prevent food shortages: (1) we need to reduce the footprint of anthropogenic activities on the planet to slow down global warming, climate change and the stresses they impose on agriculture; and (2) the resilience of crops to abiotic and biotic stresses should be augmented by means of breeding, genetic engineering, improved practices and other novel approaches.

A simplified model for the impacts of human activities on agriculture is shown in figure 1. The overall trend depicted in the figure is a complex trend in which the human population increases, and with it the demand for agricultural food production rises. However, human expansion is also accompanied by a decrease in available agricultural land and water resources, and the introduction of multiple pollution sources into our air, soil and water, collectively causing multiple types of abiotic and biotic stresses that reduce agricultural productivity. The two possible avenues that could resolve this conflict (between the increased demand for food supply and the collective stresses that anthropogenic activities inflict on agriculture) are as follows: (i) reduce the negative impacts of human activity on our planet (which to date we are largely failing to do), and (ii) increase the resilience of crops to the different stresses that anthropogenic activities inflict on them (i.e. the ‘Resilience Revolution’), which is the subject of this Theme Issue.

## 2. Worsening conditions and the absence of action: where will they lead us?

At the current rate of greenhouse gas emissions, global warming is projected to increase global average temperatures by another 1.2°C by 2050−2060 (to 2.7°C above pre-industrial temperatures). While this increase may not be alarming to many, what is important to remember is that such an increase will be accompanied by much more extreme conditions in different parts of our planet. For example, the number of days per year during which the temperature will exceed 35°C is expected to be fourfold higher than pre-industrial levels in some parts of Europe. Additional expected changes in the weather pattern are an increase in the intensity and number of storms and flooding/waterlogging events or droughts, depending on geographic location. Tropospheric levels of ozone (O_3_), microplastics and other pollutants are also expected to significantly increase, with potential negative implications for agricultural productivity and safety. A myriad of other negative factors are also expected to increase; however, given the high sensitivity of plant reproductive processes (needed for grain production/yield in rice, wheat, corn, soybean and many other crops) to heat stress, the simple increase in the number of consecutive days above a temperature threshold could be the most damaging factor to agricultural production worldwide. Thus, higher day and night temperatures occurring during fertilization, seed set and/or seed maturation/filling over the next 50−100 years could have a devastating impact on agricultural productivity and yield [15]. The effects of high temperature on yield could also be worsened by conditions of water deficit (at different levels of drought) or waterlogging (following floods), as these conditions prevent or suppress the ability of plants to cool themselves via transpiration [16]. Many of the worsening environmental conditions described above are also predicted to cause about 50% reduction in arable land area by 2050, further limiting agricultural productivity [17]. Although it is hard to predict where we will be in 50−100 years, if we do not act now, we could face a global decrease in food supply that will cause a food shortage crisis for our growing population (figure 1). For more detailed and critical reviews of these subjects, the reader is referred to [18–21] in this issue.

## 3. How should we study abiotic stress in plants if we want to succeed?

The traditional approach of scientific studies to date is to identify a problem in the field and/or nature (e.g. heat stress) and study it in the laboratory/growth chamber/greenhouse under controlled conditions (typically using the ‘reductionist’ approach of controlling all other conditions except for the one under study, e.g. heat stress). Following studies in the lab and testing in the greenhouse and/or growth chamber, the developed crops (with enhanced resilience under laboratory/growth chamber/greenhouse conditions) are then tested in the field (and then back to the lab for more improvements, i.e. the ‘lab-to-field-to-lab’ method). By contrast, breeders have traditionally been conducting their work in the field, in which a myriad of conditions and stresses occur and constantly change. The relative success of breeders in generating stress-resilient crops, compared with scientists in the laboratory, is rather obvious, suggesting that future scientific initiatives should focus on studying crops within their natural environment (i.e. the field). Thus, instead of the ‘lab-to-field-to-lab’ method, we should rather use a ‘field-to-lab-to-field’ approach (i.e*.* studying/sampling in the field, analysing in the laboratory and then directly testing in the field) that will consider the multiple abiotic and biotic conditions that occur in the field [11,22]. Alternatively, controlled environmental chambers, rooms or large indoor structures capable of mimicking the current and future outdoor conditions of the field (‘field simulators’) could be used. High-tech phenotyping abilities applied to indoor controlled, or field, conditions could also allow scientists to create dynamic models of growth responses to different stress/multi-stress conditions that will predict how different genotypes will perform in the field under different conditions and soil types, and these models will also help us to design stress-resilient crops with stable yield. The impact of agricultural management practices, e.g. cover crops, crop rotations, the use of organic fertilizers/weed control/insecticides and bio- and chemo-stimulants, should also be integrated into the studies/analyses described above to reflect the ‘real’ conditions in the field (e.g. [2,3,5,23–25]).

Using the ‘field-to-lab-to-field’ (a more ‘holistic’ approach), coupled with advanced technologies, could therefore allow us to truly understand the response of crops to different stress conditions that occur in the field [11,22]. The advent of machine learning- and artificial intelligence-driven computational tools [26] would be key in untangling the different responses of crops identified under real or simulated field conditions—for example by using multiple omics platforms coupled with phenotyping and physiological analysis—and allow us to pinpoint the critical molecular/biochemical/physiological ‘weak links’ in each crop that need to be strengthened by means of breeding and/or CRISPR (Clustered Regularly Interspaced Short Palindromic Repeats)-driven genetic modifications.

## 4. What should we focus on to enhance resilience?

Many molecular targets or ‘weak links’ that need to be strengthened in crops (to increase resilience) are currently being investigated in many different laboratories worldwide. These include strengthening crop resilience to heat stress, drought, salinity, cold stress, freezing, insects and different pathogens. Some excellent examples of these studies are discussed elsewhere in this Theme Issue (e.g. [21,24,27–37]). Many of the current studies of crop resilience to stress are however focused on specific time points or developmental stages/tissues (e.g. seedlings or vegetative tissues of mature crops) and do not address the impacts of stress on crop reproductive processes. While vegetative growth supports reproduction and produces biomass, plant reproduction is critical for seed production, which is the major component in the yield of our main grain crops, including rice, wheat, corn and soybean [15]. Importantly, recent molecular studies, as well as past breeding efforts, have revealed that the molecular mechanisms that contribute to the resilience of vegetative tissues to stress may not be similar to those that contribute to the resilience of reproductive tissues [16]. A key example for such ‘differential’ resilience between vegetative and reproductive tissues is heat stress in plants. In most plants, temperatures that will arrest plant reproduction have a minimal impact on vegetative growth—or they even enhance it. It is also now well established that photosynthesis, transpiration and water loss are not limited to leaves and that considerable non-foliar photosynthesis takes place [38]. Non-foliar photosynthesis, transpiration and water loss could also be differentially influenced by changing environmental conditions [39]. More efforts are therefore needed to determine the impact of different stresses on crops at different developmental stages, as well as on different vegetative and reproductive tissues of crops, and special attention should be given to strengthening the resilience of reproductive processes to different types of stress, as well as to combinations of stresses[40].

Another overall change that may be needed in scientists' approach to hardening crops to climate change is that we should stop thinking that we know what will work based on all past research and knowledge (that was mainly obtained using the ‘reductionist’ approach, as described above). We should simply focus on what really works in the field, based on breeders’ success, the success of different types of invasive species and weeds, and the tolerance of wild crop cultivars to different stresses. If we let nature guide us in identifying what works, we may have better success in developing crops with heightened resilience to climate change (e.g. [11,21,22,35,37]). Nature is a master of finding solutions through evolution, and evolution can happen quickly and provide insights into how plants adapt to climate change.

Improving or manipulating the plant and soil microbiomes is an emerging concept that can also help in mitigating the impacts of climate change on crops (e.g. [23,41]). This is an especially critical issue as multiple global change factors acting together have been shown to reduce the diversity and function of the soil microbiome, with potential negative impacts on crops [9].

While improving the resilience of crops to climate change, we should also consider lowering agriculture’s current footprint on greenhouse gas accumulation in our atmosphere. Thus, efforts should be made to generate crops that produce less greenhouse gasses (for example less NO) and at the same time sequester more CO_2_ into the soil [2,3]. These features should be bred into our elite cultivars together with heightened resilience to stress, to improve the overall capacity of crops to fight global warming and climate change. In addition, the use of regenerative agriculture practices should be adopted by more farmers and growers.

## 5. Do we have enough time?

This could unfortunately be a life-or-death question for many of us, especially for the poor who live in areas where crop productivity is already challenged and/or where changes to environmental conditions have already caused extreme events that reduced yield. Is there sufficient time to develop crops that can withstand the new and much harsher conditions that are anticipated in the not-so-distant future? Time is required not only to understand the processes and underlying mechanisms that we can manipulate to produce more resilient crops and generate them (efforts that would need significantly more funding and resources), but also to allow for the regulation processes required before introducing genetically modified crops into our agricultural systems. Shortening the regulatory processes (that are currently severely limiting the pace at which genetically modified resilient crops can be introduced into our agricultural systems) would require global support and collaboration from and between governments and big biotech and seed companies. In addition, the richer ‘Global North’ would need to consider the problems, challenges and needs of the poorer ‘Global South’, even if they are not necessarily profitable (a critical question given the different types of impacts that climate change may have on different geographical locations). Currently—and considering the rapid pace at which anthropogenic activities are already limiting agricultural productivity—there appears to be insufficient investment from governments and the private sector in hardening agriculture to withstand climate change, as well as an insufficient amount of collaboration across countries and continents. A drastic improvement in funding, coupled with enhanced international collaborations (that include academia, governments and industry), the opening of dedicated research centres/institutes and reduced regulation on the introduction of genetically modified crops into our fields are desperately needed if we want to succeed in this grand challenge [2,3]. Climate change is perhaps the defining crisis of our time, and we need drastic and timely measures to address and solve it.

## 6. Integrating breeding with genetic engineering, with…?

If we want to solve the grand challenge of producing enough food for a growing population under the strain of global warming and climate change (with less arable land and freshwater resources; figure 1), we need to keep our minds open to integrating many different disciplines and research areas into agriculture. For example, material sciences and chemistry/physics could offer solutions in the form of smart nanoparticles, polymers and other materials that will alleviate heat stress or high radiation, store water and nutrients, detoxify fields and/or fight pathogen/insect outbreaks. Engineering, drones/satellites, hyperspectral imaging and robotics could offer the prospect of smart farming/agriculture and weed control. High-tech state-of-the-art growth facilities capable of replicating the real field conditions anywhere in the world today, as well as the predicted future environment of our fields, are required if we are to truly understand the underlying mechanisms of resilience to multiple stressors required to produce tomorrow’s crops today. Artificial intelligence and machine learning [26] could also facilitate the development of such new and novel tools, as well as help in identifying critical phenotype–genotype associations important for hardening crops in the field. Improving and developing new methods for sustainable agriculture (using the above-described multidisciplinary approaches) and being able to recycle and re-use resources (for example using biostimulants to increase the nutrient and water use efficiency of crops and recycle waste organic products, as well as different regenerative agriculture practices to decrease waste and greenhouse gas production) are also important aspects that need to be considered. Keeping an open mind and enhancing cross-disciplinary collaboration are therefore likely to considerably improve our chances at mitigating the impacts of climate change on agriculture (e.g. [20,23,25,27,29,34–37,40,42,43]).

## 7. Stress combination as a special challenge

One of the negative ‘side effects’ of the ‘reductionist’ approach that was traditionally used to study plant stress resilience at the molecular level in the past is the lack of knowledge/understanding of what truly happens under field conditions [11,22]. In the field, crops are routinely subjected to a combination of multiple stressors. For example, elevated temperatures in the middle of the day could happen in combination with conditions of low or moderate levels of water deficit, nutrient imbalance, and other factors such as high ozone levels and/or pathogen/insect attack. As was demonstrated over 20 years ago with drought and heat [44], the response of plants to a combination of two different abiotic stresses cannot be predicted from simply summing up the effects of each of these individual stresses on plants [11,22,40,42,45]. The state of ‘stress combination’ should therefore be considered as a new type of stress that requires a new type of acclimation response (as reviewed in this issue by [40], describing the process of ‘differential transpiration’ under conditions of drought and heat stress combination in crops, as well as by [33] and [36] addressing autophagy and molecular integration of acclimation pathways during stress combination, respectively). As climate change and global warming are expected to increase the frequency and intensity of stress combination events (e.g. droughts or waterlogging stresses combined with heat waves, or droughts occurring in areas with high salinity under high or low temperatures), stress combination needs to be studied in different crops around the world and resilience to stress combination should be an important target for enhancing plant resilience to climate change.

## 8. Summary

The worsening of environmental conditions on our planet, coupled with the increased demand for food (driven by our population increase) and dwindling of resources, puts a severe strain on our agricultural systems (figure 1). As the pace of climate change and global warming appears to have accelerated in the past 10−15 years and attempts to slow these processes are stalling or largely unsuccessful, urgency is needed in trying to enhance the resilience of our major crops to the unique and emerging stressful conditions they will face in the not-so-distant future. As the ‘green revolution’ allowed us to improve yield and prevent hunger, we now need the ‘Resilience Revolution’ to allow our crops to withstand the environmental changes that are to come. Although the task is daunting, some success stories exist (e.g. the enhancement of flooding tolerance in rice through a combination of molecular genetics and breeding; [46]). However, time is short, and we must act now!

We hope that this Theme Issue will increase the awareness of the general scientific community, decision makers worldwide and the public to the threats that global warming and climate change pose to agriculture and the supply of food needed to support our growing population.

## Data Availability

This article has no additional data.

## References

[1] IPCC, Core Writing Team. 2023 Summary for policymakers. In Climate change 2023: synthesis report. contribution of working groups I, II and III to the sixth assessment report of the intergovernmental panel on climate change (eds H Lee, J Romero), pp. 1–34. Geneva, Switzerland: IPCC. (10.59327/IPCC/AR6-9789291691647.001)

[2] Rhee SY *et al*. 2025 Resilient plants, sustainable future. Trends Plant Sci. **30**, 382–388. (10.1016/j.tplants.2024.11.001) 39643496

[3] Hirt H *et al*. 2023 PlantACT! - how to tackle the climate crisis. Trends Plant Sci. **28**, 537–543. (10.1016/j.tplants.2023.01.005) 36740490

[4] Richardson K *et al*. 2023 Earth beyond six of nine planetary boundaries. Sci. Adv. **9**, eadh2458. (10.1126/sciadv.adh2458) 37703365 PMC10499318

[5] Zandalinas SI, Fritschi FB, Mittler R. 2021 Global warming, climate change, and environmental pollution: recipe for a multifactorial stress combination disaster. Trends Plant Sci. **26**, 588–599. (10.1016/j.tplants.2021.02.011) 33745784

[6] Rillig MC, Ryo M, Lehmann A. 2021 Classifying human influences on terrestrial ecosystems. Glob. Chang. Biol. **27**, 2273–2278. (10.1111/gcb.15577) 33660892

[7] Sage RF. 2020 Global change biology: a primer. Glob. Chang. Biol. **26**, 3–30. (10.1111/gcb.14893) 31663217

[8] Bi M, Li H, Meidl P, Zhu Y, Ryo M, Rillig MC. 2024 Number and dissimilarity of global change factors influences soil properties and functions. Nat. Commun. **15**, 8188. (10.1038/s41467-024-52511-2) 39294171 PMC11410830

[9] Rillig MC, Ryo M, Lehmann A, Aguilar-Trigueros CA, Buchert S, Wulf A, Iwasaki A, Roy J, Yang G. 2019 The role of multiple global change factors in driving soil functions and microbial biodiversity. Science **366**, 886–890. (10.1126/science.aay2832) 31727838 PMC6941939

[10] Lesk C, Anderson W, Rigden A, Coast O, Jägermeyr J, McDermid S, Davis KF, Konar M. 2022 Compound heat and moisture extreme impacts on global crop yields under climate change. Nat. Rev. Earth Environ. **3**, 872–889. (10.1038/s43017-022-00368-8)

[11] Mittler R. 2006 Abiotic stress, the field environment and stress combination. Trends Plant Sci. **11**, 15–19. (10.1016/j.tplants.2005.11.002) 16359910

[12] Alizadeh MR, Adamowski J, Nikoo MR, AghaKouchak A, Dennison P, Sadegh M. 2020 A century of observations reveals increasing likelihood of continental-scale compound dry-hot extremes. Sci. Adv. **6**, z4571. (10.1126/sciadv.aaz4571) PMC7531886 32967839

[13] Long SP, Marshall-Colon A, Zhu XG. 2015 Meeting the global food demand of the future by engineering crop photosynthesis and yield potential. Cell **161**, 56–66. (10.1016/j.cell.2015.03.019) 25815985

[14] Lesk C, Rowhani P, Ramankutty N. 2016 Influence of extreme weather disasters on global crop production. Nature **529**, 84–87. (10.1038/nature16467) 26738594

[15] Sadok W, Jagadish SVK. 2020 The hidden costs of nighttime warming on yields. Trends Plant Sci. **25**, 644–651. (10.1016/j.tplants.2020.02.003) 32526169

[16] Sinha R, Zandalinas SI, Fichman Y, Sen S, Zeng S, Gómez‐Cadenas A, Joshi T, Fritschi FB, Mittler R. 2022 Differential regulation of flower transpiration during abiotic stress in annual plants. New Phytol. **235**, 611–629. (10.1111/nph.18162) 35441705 PMC9323482

[17] Hassani A, Azapagic A, Shokri N. 2020 Predicting long-term dynamics of soil salinity and sodicity on a global scale. Proc. Natl Acad. Sci. USA **117**, 33017–33027. (10.1073/pnas.2013771117) 33318212 PMC7776813

[18] Long S. 2025 Needs and opportunities to future-proof crops and the use of crop systems to mitigate atmospheric change. Phil. Trans. R. Soc. B **380**, 20240229. (10.1098/rstb.2024.0229) 40439295 PMC12121382

[19] Ainsworth E, Sanz-Saez A, Leisner C. 2025 Crops and rising atmospheric CO₂ – friends or foes? Phil. Trans. R. Soc. B **380**, 20240230. (10.1098/rstb.2024.0230) 40439307 PMC12121383

[20] Foyer C, Wang A, Shi K. 2025 CO₂ signalling in plants. Phil. Trans. R. Soc. B **380**, 20240247. (10.1098/rstb.2024.0247) 40439310 PMC12121397

[21] Cagnola J, Rotili DH, Otegui ME, Casal JJ. 2025 50 years of breeding to improve yield: how maize stands up to climate change. Phil. Trans. R. Soc. B **380**, 20240250. (10.1098/rstb.2024.0250) 40439315 PMC12121388

[22] Mittler R, Blumwald E. 2010 Genetic engineering for modern agriculture: challenges and perspectives. Annu. Rev. Plant Biol. **61**, 443–462. (10.1146/annurev-arplant-042809-112116) 20192746

[23] Brito-Lopez C, van der Wielen N, Barbosa M, Karlova R. 2025 Plant-growth promoting microbes and microalgae-based biostimulants: sustainable strategy for agriculture and abiotic stress resilience. Phil. Trans. R. Soc. B **380**, 20240251. (10.1098/rstb.2024.0251) 40439314 PMC12132076

[24] Siddique M, De Ocampo M, Bagunu E, Quick WP, Diaz MG, Henry A. 2025 Genome-wide association mapping for salinity recovery of rice seedlings grown in hydroponic and field conditions. Phil. Trans. R. Soc. B **380**, 20240248. (10.1098/rstb.2024.0248) 40439308 PMC12121376

[25] Verslues P. 2025 Understanding and optimizing plant growth in water-limited environments: ‘Growth versus defense’ or ‘Growth versus risk mitigation’? Phil. Trans. R. Soc. B **380**, 20240232. (10.1098/rstb.2024.0232) 40439302 PMC12121379

[26] Singhal R, Izquierdo P, Ranaweera T, Segura Abá K, Brown B, Lehti-Shiu M, Shiu SH. 2025 Using supervised machine-learning approaches to understand abiotic stress tolerance and design resilient crops. Phil. Trans. R. Soc. B **380**, 20240252. (10.1098/rstb.2024.0252) 40439305 PMC12121380

[27] Shumayla, Alejo-Jacuinde G, Silva-Villatoro P, Nwoko CL, Oliver M, Herrera-Estrella L. 2025 The promise of resurrection plants in enhancing crop tolerance to water scarcity. Phil. Trans. R. Soc. B **380**, 20240231. (10.1099/rstb.2024.0231) 40439304 PMC12121386

[28] Schroeder J, Kasera M, Ceciliato PHO, Lopez B, Hauser F, Gendron JM. 2025 Identification of F-box proteins in ABA- and GA-regulated seed germination: interaction of GASA1 signaling peptide and ABA-induced ubiquitination. Phil. Trans. R. Soc. B **380**, 20240233. (10.1098/rstb.2024.0233) 40439299 PMC12121378

[29] Chopra P *et al*. 2025 Priming thermotolerance: unlocking heat resilience for climate-smart crops. Phil. Trans. R. Soc. B **380**, 20240234. (10.1098/rstb.2024.0234) 40439313 PMC12121387

[30] Cavanagh A, Matthews M. 2025 The heat is on: improving the temperature response of photosynthesis to maintain crop yields in a changing climate. Phil. Trans. R. Soc. B **380**, 20240235. (10.1098/rstb.2024.0235) 40439311 PMC12121381

[31] Nakashima K, Yamaguchi-Shinozaki K, Shinozaki K. 2025 Transcriptional gene network involved in drought stress response: application for crop breeding in the context of climate change. Phil. Trans. R. Soc. B **380**, 20240236. (10.1098/rstb.2024.0236) 40439309 PMC12132078

[32] Renziehausen T, Dirr A, Schmidt-Schippers R, Flashman E, Schippers J. 2025 Oxygen sensing and plant adaptation to flooding in a changing climate. Phil. Trans. R. Soc. B **380**, 20240238. (10.1098/rstb.2024.0238) 40439298 PMC12121377

[33] Agbemafle W, Jayasinghe V, Bassham D. 2025 Can autophagy enhance crop resilience to environmental stress? Phil. Trans. R. Soc. B **380**, 20240245. (10.1098/rstb.2024.0245) 40439312 PMC12121398

[34] Lim SD, Lomas JS, Islam M, Pérez López AV, Kim S-h, Petrusa LM, Yim WC, Cushman J. 2025 Synthetic crassulacean acid metabolism (SynCAM) for improving water-use efficiency in plants. Phil. Trans. R. Soc. B **380**, 20240249. (10.1068/rstb.2024.0249) 40439297 PMC12121396

[35] Yun P, Shahzad B, Hasanuzzaman M, Islam T, Shabala L, Zhou M, Venkataraman G, Chen ZH, Shabala S. 2025 Learning from nature: photosynthetic traits conferring superior salt tolerance in wild rice Oryza coarctata. Phil. Trans. R. Soc. B **380**, 20240242. (10.1098/rstb.2024.0242) 40439303 PMC12132074

[36] Kumar V, Knieper M, Vogelsang L, Denjali I, Seidel T, Dietz KJ. 2025 Principles of signal integration in combinatorial stress acclimatization. Phil. Trans. R. Soc. B **380**, 20240243. (10.1098/rstb.2024.0243) 40439301 PMC12132075

[37] Hofmann T, Atkinson W, Fan M, Simkin A, Jindal P, Lawson T. 2025 Impact of climate driven changes in temperature on stomatal anatomy and physiology. Phil. Trans. R. Soc. B **380**, 20240244. (10.10968/rstb.2024.0244) 40439300 PMC12121385

[38] Lawson T, Milliken AL. 2023 Photosynthesis – beyond the leaf. New Phytol. **238**, 55–61. (10.1111/nph.18671) 36509710 PMC10953325

[39] Simkin AJ, Faralli M, Ramamoorthy S, Lawson T. 2020 Photosynthesis in non‐foliar tissues: implications for yield. Plant J. **101**, 1001–1015. (10.1111/tpj.14633) 31802560 PMC7064926

[40] Sinha R *et al*. 2025 The differential transpiration response of plants to stress. Phil. Trans. R. Soc. B **380**, 20240241. (10.1098/rstb.2024.0241) 40439306 PMC12121384

[41] Kozaeva E, Eida AA, Gunady EF, Dangl JL, Conway JM, Brophy JA. 2024 Roots of synthetic ecology: microbes that foster plant resilience in the changing climate. Curr. Opin. Biotechnol. **88**, 103172. (10.1016/j.copbio.2024.103172) 39029405

[42] Zandalinas SI, Mittler R. 2022 Plant responses to multifactorial stress combination. New Phytol. **234**, 1161–1167. (10.1111/nph.18087) 35278228

[43] Zandalinas SI, Peláez‐Vico MÁ, Sinha R, Pascual LS, Mittler R. 2024 The impact of multifactorial stress combination on plants, crops, and ecosystems: how should we prepare for what comes next? Plant J. **117**, 1800–1814. (10.1111/tpj.16557) 37996968

[44] Rizhsky L, Liang H, Shuman J, Shulaev V, Davletova S, Mittler R. 2004 When defense pathways collide. The response of Arabidopsis to a combination of drought and heat stress. Plant Physiol. **134**, 1683–1696. (10.1104/pp.103.033431) 15047901 PMC419842

[45] Sato H, Mizoi J, Shinozaki K, Yamaguchi‐Shinozaki K. 2024 Complex plant responses to drought and heat stress under climate change. Plant J. **117**, 1873–1892. (10.1111/tpj.16612) 38168757

[46] Xu K *et al*. 2006 Sub1A is an ethylene-response-factor-like gene that confers submergence tolerance to rice. Nature **442**, 705–708. (10.1038/nature04920) 16900200

